# Physiological Impact of Chromatic-Weight Illusions in Augmented Reality: A Comparative sEMG Analysis of Muscle Fatigue and Stability

**DOI:** 10.3390/s26092575

**Published:** 2026-04-22

**Authors:** Jun Wang, Julia Greenfield, Peter Mitrouchev, Guiqin Li, Franck Quaine

**Affiliations:** 1 Department of Mechatronics, Shanghai Jianqiao University, Shanghai 201306, China; 2 G-SCOP Lab, University Grenoble Alpes, CNRS, 38000 Grenoble, France; 3 Institut für Digitale Medizin, Philipps University of Marburg, 35037 Marburg, Germany; 4 Shanghai Key Laboratory of Intelligent Manufacturing and Robotics, Shanghai University, Shanghai 200072, China; 5 GIPSA Lab, University Grenoble Alpes, CNRS, 38000 Grenoble, France

**Keywords:** muscle co-contraction, muscle fatigue, augmented reality, color–weight illusion

## Abstract

During manual operations, the human brain relies on mediated visual stimuli such as color to estimate an object’s weight and adjust muscle force through the central nervous system (CNS). This study examines the neuromuscular “reality gap” induced by the color–weight illusion (CWI) during repetitive lifting tasks in an augmented reality (AR) interface. We analyzed the median frequency (MDF) and Co-Contraction Index (CCI) of the biceps and triceps muscles to quantify physiological strain under varying luminance conditions in both AR and physical environments. The results reveal that AR significantly amplifies the CWI, with black stimuli triggering an aggressive joint-stiffening strategy in the AR group (APG). Compared with the physical reality group, the AR group showed lower overall endurance (91.4 ± 22.8 vs. 100.1 ± 12.5 repetitions) and a stronger physiological response to the black stimulus. In the AR group, the black condition was associated with a terminal CCI of 84.7 ± 25.4% and an MDF decline of approximately 21.7 Hz, whereas the corresponding contrast was attenuated in the physical reality group. These findings demonstrate a critical decoupling between behavioral output and internal physiological strain, indicating that the CNS treats virtual visual cues as high-reliability signals that increase metabolic “bio-cost” despite task completion parity. This research identifies a “masking effect” where behavioral metrics hide severe ergonomic risks, providing novel approaches for managing musculoskeletal health in industrial settings and personalizing coordination training in clinical rehabilitation.

## 1. Introduction

Augmented reality (AR) has established itself as a transformative technology for enhancing human motor capabilities in high-precision sectors such as industrial assembly and robotic-assisted rehabilitation [1,2]. By superimposing digital information onto the physical world, these systems allow for the real-time modulation of human–computer interaction through targeted sensory-mediated cues [3]. A core research focus within this domain is the manipulation of weight perception, where visual markers are utilized to invoke cross-modal phenomena such as the color–weight illusion (CWI) [4,5]. However, a significant scientific hurdle remains the “reality gap,” which describes the persistent discrepancies in sensory integration and physiological behavior observed between virtual and physical environments [6]. While previous investigations have documented behavioral shifts in response to AR-mediated visual stimuli, the underlying neuromuscular strategies governing these responses remain poorly understood [1,5]. In particular, evidence is lacking to explain why digital visual cues can trigger significant physiological exertion that does not occur under identical physical conditions. This deficiency in mechanistic understanding prevents the development of a unified perception–action model for AR, which is essential for ensuring user safety and preventing musculoskeletal fatigue in immersive environments.

Substantial research has explored the use of digital overlays to modulate human perception and motor performance [7,8]. Initial investigations in this field focused on the integration of visual and haptic cues, demonstrating that virtual modifications can successfully induce weight illusions [5,9]. Subsequent studies applied these principles to AR environments to enhance physical endurance. Notably, early work by Ban et al. reported that altering the visual brightness of objects could delay the onset of subjective fatigue and increase the total number of lifting repetitions [10]. Such findings suggested that digital cues could effectively optimize human effort during manual tasks. Despite this progress, the current understanding of these effects is limited by a significant theoretical contradiction. Most existing studies attribute the increase in endurance to a reduction in muscle force output. However, this explanation is inconsistent with Newtonian mechanics, as changing the color of an object does not alter its physical mass or the net torque required for movement [11]. Furthermore, previous research has primarily focused on external behavioral outcomes while neglecting the internal neuromuscular strategies that govern joint stability. A critical knowledge gap exists regarding how the brain coordinates antagonistic muscle pairs to manage the sensory conflict between virtual expectations and physical feedback. Specifically, it remains unclear whether the observed behavioral changes in AR are driven by shifts in muscle co-contraction rather than changes in net motor output.

The primary objective of this research is to elucidate the neuromuscular mechanisms that govern the “reality gap” during AR-mediated manual tasks. We specifically investigate whether the CWI modulates the internal coordination of antagonistic muscle pairs rather than the net force output. Based on sensory re-weighting theory, the brain dynamically adjusts its reliance on different sensory channels depending on their perceived reliability [12]. In AR environments, the head-mounted display (HMD) limits natural haptic sensitivity and peripheral vision, potentially causing the nervous system to prioritize digital visual cues over physical feedback [13,14]. Furthermore, according to impedance control theory, the brain may increase joint stiffness through muscle co-contraction to manage the uncertainty caused by visuo-haptic mismatches [15,16]. Consequently, we conducted a controlled experiment to compare upper-limb motor strategies in both a no-HMD physical environment and an AR-mediated space. We recorded surface electromyography (sEMG) to calculate the Co-Contraction Index (CCI) and the median frequency (MDF) of the biceps and triceps while tracking three-dimensional kinematics to monitor movement execution and define movement timing during the task. We tested three primary hypotheses: (1) the CWI alters the internal muscle co-contraction strategy without changing the net motor output required for the movement; (2) the physiological effect of the illusion is significantly amplified in the AR environment compared to the physical baseline due to increased visual weighting; and (3) the elevated co-contraction in AR leads to a measurable acceleration in localized muscle fatigue during repetitive lifting.

The significance of this research lies in its transition from observing behavioral outcomes to defining the internal neuromuscular strategies that constitute the “reality gap.” By clarifying that virtual luminance modulates joint stiffness through antagonistic co-contraction—rather than altering net force output—this work provides a specific biomechanical explanation for how AR-mediated illusions increase the metabolic demand of physically identical tasks. The proposed methodology establishes a benchmarking framework for evaluating the “physiological transparency” of AR overlays, ensuring that digital instructions do not inadvertently trigger energy-inefficient motor strategies. Practically, these findings empower ergonomic designers to mitigate sub-clinical fatigue in manual laborers by selecting optimal luminance levels for virtual markers in industrial assembly. Furthermore, the strategic use of brightness-driven co-contraction offers a non-mechanical tool for modulating joint stability during robotic-assisted rehabilitation and stability training. The remainder of this paper is organized as follows: Section 2 details the experimental materials and methodological procedures. Section 3 presents the quantitative results regarding co-contraction indices and spectral frequency shifts. Section 4 provides a comprehensive discussion of sensory re-weighting and its ergonomic implications. Finally, Section 5 summarizes the conclusions and suggests directions for future work.

## 2. Materials and Methods

### 2.1. Participants

Twenty-five healthy volunteers participated in this study. The participants were assigned to two independent groups based on the experimental environment: the augmented reality group (ARG) and the physical reality group (PRG). The ARG consisted of fifteen subjects (10 males and 5 females; age: 21 ± 7 years; height: 1.70 ± 0.06 m; weight: 64.0 ± 7.9 kg) who performed the lifting task in the AR environment. The PRG included ten subjects who completed the identical protocol in a natural physical setting without a head-mounted display (HMD). All participants were right-handed and had no history of musculoskeletal or neurological impairments [17,18]. We screened all volunteers to ensure they had normal or corrected-to-normal vision. To minimize cognitive bias, we blinded the participants to the specific purpose of the color manipulation [10]. No subjects had prior experience with augmented reality systems. The research protocol received approval from the Ethics Committee of Shanghai Jianqiao University (Number: SJQU20240326). Every participant provided written informed consent before data collection.

### 2.2. Experimental Instrumentation

**Figure 1:**
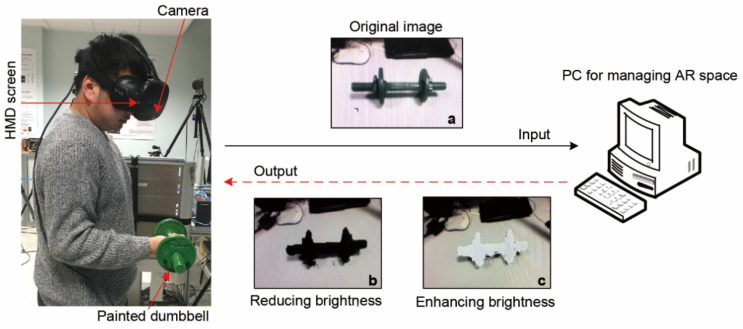
Schematic diagram of the algorithm: (**a**) original image; (**b**,**c**) modified images.

**Figure 2:**
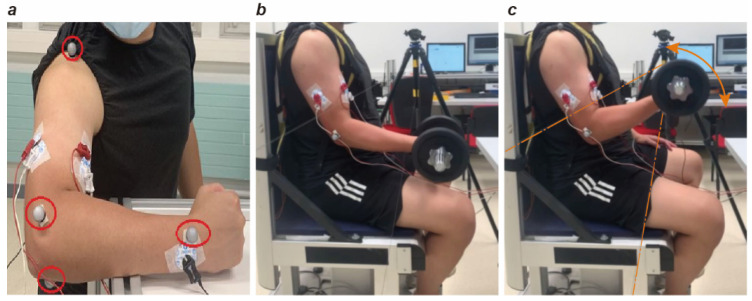
Experimental preparation: (**a**) Electrode and marker positions on the arm. Markers are circled in red, while electrodes are connected to black and red clips. (**b**) Customized chair: keeping the upper body strapped and the back straight. (**c**) Range and direction of movement shown by the double-headed arrow.

We employed a synchronized multimodal sensing platform to capture biomechanical and physiological data:

- Kinematics: A twelve-camera infrared motion capture system (Qualisys Oqus 6+, Göteborg, Sweden) tracked movement at 1 kHz. Four reflective markers were placed on the dominant arm of the subjects according to [19]. The markers were positioned at the following locations: clavicle–acromion joint (CAJ), humerus lateral epicondyle (HLE), humerus medial epicondyle (HME), and radius styloid process (RSP) [20].
- AR System: We used an HTC Vive head-mounted display (HTC Corp., Taoyuan, China) operating in video see-through mode to create the immersive environment. A custom Unity (Version [2022LTS]) 3D-based pipeline was developed to modify the apparent surface brightness of the dumbbell in real time. The brightness manipulation was implemented as an absolute adjustment. The display brightness setting was fixed at “bright”, and the rendering frame rate was maintained at 30 fps throughout the experiment. All participants used the same HMD configuration. No additional manual realignment of the virtual overlay was performed before each experiment, and all AR trials were conducted under constant indoor lighting conditions. End-to-end system latency was not independently quantified in the present study (Figure 1).
- Electromyography (sEMG): We recorded surface electromyography (sEMG) signals using a Biopac MP160 system (Biopac Systems Inc., Goleta, CA, USA). The system differentially amplified the signals and sampled them at a frequency of 1 kHz. We placed the ground electrode on the ulna styloid process. Before attaching the electrodes, we shaved and cleaned the participant’s skin to reduce impedance. We used circular, self-adhesive bipolar pairs of disposable surface electrodes with a diameter of 10 mm. The center-to-center spacing between the electrodes was 10 mm. Following the SENIAM guidelines [18], we positioned the electrodes on the long head of the biceps brachii (BB) and the lateral head of the triceps brachii (TB). In this setup, the BB represented the elbow flexors, and the TB represented the elbow extensors (Figure 2a). We selected the long head of the BB because it contributes significantly to elbow flexion [21]. This selection also aligns with the experimental protocol established by Ban et al. [10]. For the TB, we specifically chose the lateral head because it directly assists in elbow extension. Previous research has also shown that the lateral head produces high levels of muscle activity during lifting tasks [22]. Therefore, this muscle head served as an ideal target for isolating the neuromuscular response in our investigation.
- Force Measurement: We used a custom crossbar embedded with two triaxial force transducers (Kistler 9047C, Winterthur, Switzerland) to measure maximal isometric forces.

### 2.3. Experimental Design and Protocol

**Figure 3:**
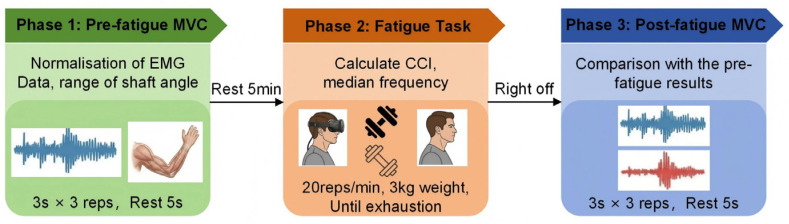
Experimental protocol.

As shown in Figure 3, the protocol followed three steps: (1) pre-fatigue maximum voluntary contraction (MVC), (2) the repetitive lifting fatigue task (FT), and (3) post-fatigue MVC. Environment (AR vs. physical) was treated as a between-subject factor, whereas color (black vs. white) was administered as a repeated condition within each environment. The order of the two color conditions was randomized across participants.

#### 2.3.1. Isometric MVC

Participants were seated in a customized chair that immobilized the upper body to maintain a straight back and prevent compensatory movements. The dominant elbow joint was positioned at 90° of flexion, with the forearm horizontal and the wrist in a neutral (0°) position. During the isometric MVC phase, participants held the force crossbar with a supinated wrist and performed three maximal contractions (3 s each) in both the flexion (pulling) and extension (pushing) directions. A 5 s rest interval was provided between successive efforts. The dynamic EMG signals recorded during the fatigue task were normalized to the peak RMS value obtained during the pre-fatigue isometric MVC trials for each muscle.

#### 2.3.2. Repetitive Lifting Fatigue Task

For the FT, participants remained in the customized chair. Shoulder fixation was strictly enforced using straps to prevent compensatory strategies, such as trunk flexion or extension, which might otherwise mitigate localized arm fatigue (Figure 2b,c). Participants in the ARG wore the HMD, while those in the PRG executed the task with natural vision.

Participants were instructed to repeatedly lift a physical dumbbell weighing 3 kg (selected based on pilot testing to ensure an appropriate sub-maximal load). The lifting motion ranged from thigh level to a target height corresponding to 30° of elbow flexion (i.e., a functional range of motion from 90° to 30°). The target height was physically indicated by a transparent fishing line; contact with the line signaled the completion of the concentric flexion phase (Figure 2b).

Movement cadence was externally paced by an auditory metronome at 20 repetitions per minute (0.33 Hz), ensuring each complete flexion–extension cycle lasted exactly 3 s. Prior to data collection, participants rehearsed the motion at a sub-fatiguing volume to familiarize themselves with the required range and tempo. The FT was continuously monitored and formally terminated by the experimenter when the participant could no longer complete the full range of motion or adhere to the auditory tempo due to volitional exhaustion.

### 2.4. Data Processing and Feature Extraction

**Figure 4:**
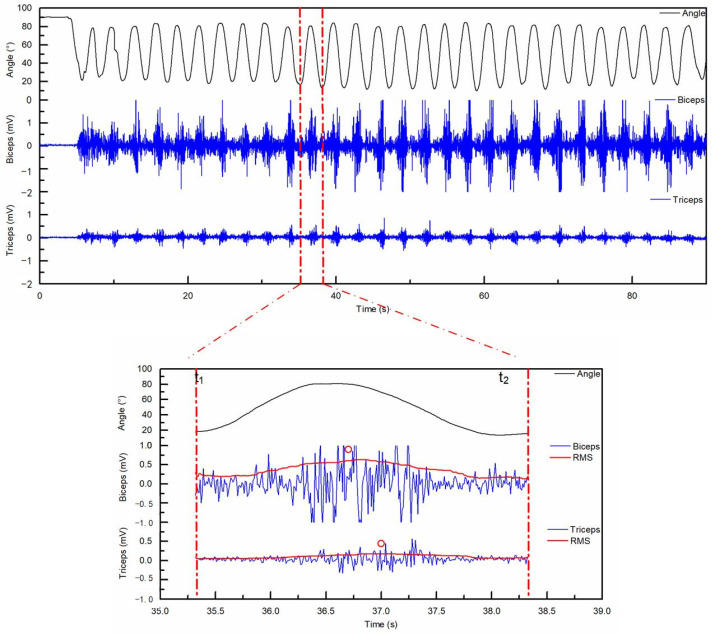
Example of time histories from one subject. Upper graph: the elbow angular position and raw EMG signal of BB and TB during FT (vertical lines indicate the one lift movement). Lower graph: the elbow angular position, raw EMG signal, and RMS in one movement. Red vertical center lines indicate the start (*t*_1_) and end (*t*_2_) time in formula (3), denoting the period of muscle contraction within one lift movement. Two red circles denote the maximum values of RMS in extension and flexion, respectively. Additional representative filtered sEMG traces and RMS envelopes for the black and white conditions are provided in Supplementary Figure S1.

We processed all kinematic, force, and EMG data using custom scripts in MATLAB (Version 9.13, MathWorks, Natick, MA, USA). The system applied a low-pass filter (12.5 Hz) to the kinematic and force signals to eliminate high-frequency noise. We processed the raw EMG signals using a band-pass filter (20–400 Hz). We also removed the DC offset and performed full-wave rectification to obtain the linear envelopes [18]. We identified the start *t*_1_ and end *t*_2_ of each lifting movement by analyzing the minimum elbow flexion angle (Figure 4).

#### 2.4.1. EMG Spectral Analysis (MDF)

We evaluated localized muscle fatigue by calculating the median frequency (MDF) of the EMG signals [23,24]. We applied a Fast Fourier Transform (FFT) to determine the power spectral density for each lifting cycle. The decrease in MDF served as the primary indicator of physiological exhaustion. We also compared the MDF values obtained during the MVC tests before and after the fatigue task (FT) for each color condition. To improve the transparency of the signal-processing workflow, Supplementary Figure S1 presents representative band-pass-filtered sEMG traces of the biceps brachii and triceps brachii under the black and white conditions, together with the corresponding RMS envelopes.

#### 2.4.2. EMG Amplitude and Normalization

We calculated the muscle activation level as the root mean square (RMS) of the EMG linear envelope [25]. RMS was computed using a 200 ms moving window for both MVC and task EMG signals. For normalization, the highest RMS value identified during the pre-fatigue MVC trials was defined as the reference value for each muscle.

(1) $$RMS=\sqrt{\frac{1}{N}\sum_{i=1}^{N}x_{i}^{2}}$$

In Equation (1), *x_i_* is the *i*th sample of a signal, and *N* is the number of samples in the epoch. All EMG signals have been processed using the RMS.

#### 2.4.3. EMG Antagonist-Agonist Co-Contraction Index

Muscle co-contraction was estimated using the method developed by Falconer and Winter [26,27]. This technique quantifies the simultaneous activation of muscle groups during dynamic movements. This approach assumes that the EMG amplitude reflects the relative moments of the agonist and antagonist muscles. We calculated the Co-Contraction Index (CCI) from the normalized muscle activation signals during each lift [28]. The calculations used the following equations:

(2) $$I_{total}=\int_{t_{1}}^{t_{2}}[EMG_{flexors}(t)+EMG_{extensors}(t)]dt$$

(3) $$CCI=\frac{2\cdot \int_{t_{1}}^{t_{2}}EMG_{antagonist}(t)dt}{I_{total}}\cdot 100\%$$

In these formulas, *EMG_extensors_* represents the activation of the triceps (TB), and *EMG_flexors_* represents the recorded activation of the biceps (BB). The variables *t*_1_ and *t*_2_ denote the period of muscle contraction within one lift cycle based on the elbow angle.

### 2.5. Statistical Analysis

All statistical analyses were performed using linear mixed-effects models (LMMs) in the R software environment (version 4.0.2) [29]. Data distribution was assessed using the Shapiro–Wilk test prior to inferential analysis, and the relevant variables were found to be approximately normally distributed. This study was designed as an exploratory investigation, and no formal a priori power analysis was reported. Therefore, the present findings should be interpreted with appropriate caution. Linear mixed-effects models (LMMs) were adopted to analyze the neuromuscular and behavioral data [30]. LMMs were adopted because they retain all available observations, account for inter-subject variability, and are appropriate for repeated-measures data with unequal group sizes. Nevertheless, the imbalance between the ARG (*N* = 15) and PRG (*N* = 10) may reduce statistical precision, especially for interaction terms and between-group comparisons [6,31]. Individual variations in the number of repetitions completed during the fatigue task were further accounted for within the LMM framework. This approach also accounts for the varying number of repetitions completed by each subject during the fatigue task. In the mixed-effects models, environment was modeled as a between-subject fixed factor and color as a repeated fixed factor, with subject included as a random effect [32]. For fatigue dynamics, the fatigue rate was extracted as the primary indicator. This metric was calculated by fitting a linear regression to the median frequency (MDF) time series of each participant [33,34]. The resulting regression slope was utilized to represent the rate of localized muscle exhaustion [35,36].

For non-significant findings (e.g., the behavioral metrics in the PRG), the Bayes factor (*BF*_10_) was computed to quantify the strength of evidence in favor of the null hypothesis [37]. A threshold of *BF*_10_ < 0.33 was interpreted as substantial evidence for the absence of a meaningful effect, thereby distinguishing a true null result from a mere lack of statistical power [38]. The significance level for all frequentist tests was set at *p* < 0.05.

## 3. Results

### 3.1. Behavioral Performance: Repetitive Lifting Capacity

**Figure 5:**
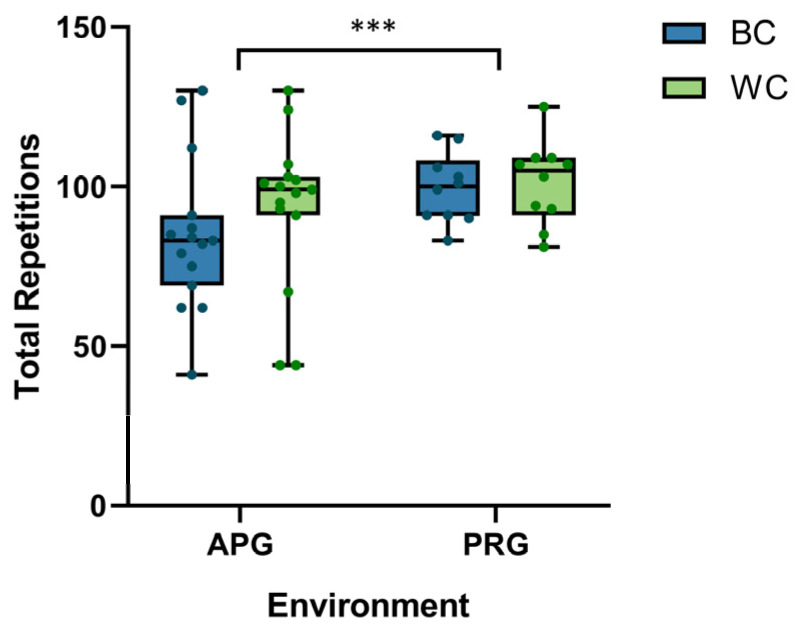
Comparative analysis of lifting endurance (total repetitions) across environments and color conditions. Individual data points represent each participant (*N__APG_* = 15, *N__PRG_* = 10), superimposed on box-and-whisker plots indicating the median and interquartile range. A highly significant main effect of environment was observed (*F*_1,23_ = 18.36, *p* < 0.001), with the physical reality group (PRG) exhibiting superior overall endurance. The effect of object color within groups did not reach statistical significance (*p* = 0.188). *** denotes *p* < 0.001.

Figure 5 illustrates the behavioral outcomes regarding the total number of repetitions performed until exhaustion. A highly significant main effect of the experimental group was identified (*F*_1,23_ = 18.36, *p* < 0.001), demonstrating that participants in the PRG possessed superior overall endurance (100.1 ± 12.5 reps) compared to those in the APG (91.4 ± 22.8 reps). As summarized in Table 1, a descriptive trend was observed within the APG, where the white color (WC) condition yielded a higher repetition count (96.7 ± 21.1) than the black color (BC) condition (86.2 ± 23.9). In contrast, endurance in the PRG remained nearly identical across color conditions (BC: 100.4 vs. WC: 99.6). While the *Environment* × *Color* interaction did not reach frequentist significance (*p* = 0.544), Bayesian analysis for the PRG yielded a *BF*__10_ = 0.28, providing substantial evidence for the null hypothesis in the physical world. This suggests that the impact of color–weight cues on physical endurance is more pronounced, descriptively, in the AR-mediated environment.

### 3.2. Indicators of Localized Muscle Fatigue: Median Frequency (MDF)

**Figure 6:**
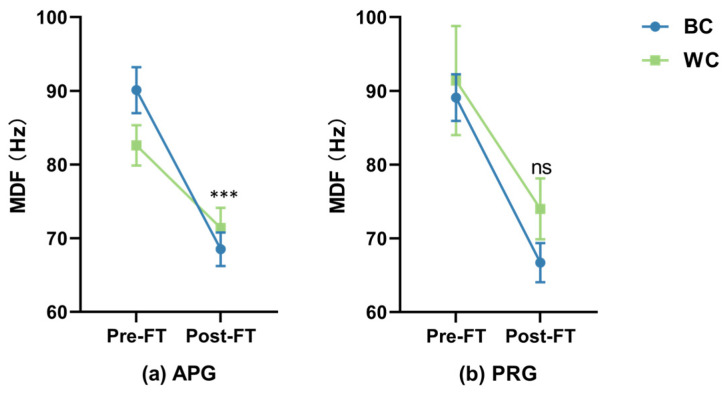
Dynamics of localized muscle fatigue illustrated by the spectral compression of the biceps brachii median frequency (MDF). The two-panel plot compares fatigue rates between the augmented reality group (**a**) and the physical reality group (**b**) from baseline (Pre-FT) to exhaustion (Post-FT). A robust *Color* × *Time* interaction was identified in the APG (*p* < 0.001), where the black stimulus induced a significantly steeper decline in MDF compared to the white stimulus. This interaction highlights how visual color cues accelerate myoelectric manifestations of fatigue. *** denotes *p* < 0.001; ns, not significant.

Figure 6 presents the progressive downward shifts in the median frequency (MDF) of the biceps brachii (BB), which serves as a primary spectral indicator of localized muscle fatigue. Following the calibration of physiological baselines, a significant main effect of object color was observed (*F*_1,92_ = 5.89, *p* < 0.001).

Crucially, as indicated by the significant interaction effect in Table 2, a robust *Color* × *Time* interaction in the LMM analysis was detected (*F*_1,92_ = 13.68, *p* < 0.001). Under the BC condition, the MDF exhibited a rapid and severe decline across both groups (APG: ∆ ≈ 21.7 Hz; PRG: ∆ ≈ 22.4 Hz), reflecting accelerated myoelectric manifestations of fatigue. Conversely, the WC condition displayed a significantly more stable spectral profile, with a much smaller decline in frequency. These results provide empirical evidence that the visual perception of “heavier” black objects directly intensifies the rate of localized exhaustion in the primary agonist muscle.

### 3.3. Neuromuscular Strategy and Joint Stability: Co-Contraction Index (CCI)

**Figure 7:**
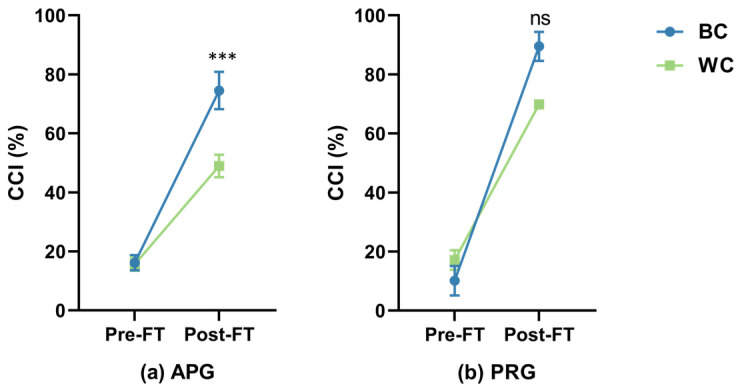
Evolution of neuromuscular coordination strategies during the fatiguing task, quantified via the Co-Contraction Index (CCI). The two-panel plot demonstrates the “reality gap” in joint-stiffening strategies. In the augmented reality group (**a**), a significant difference in CCI was observed at exhaustion between color conditions (*p* < 0.001), with the black stimulus inducing a dramatically higher synergistic activation of antagonist muscles. In contrast, the physical reality group (**b**) showed no significant difference between BC and WC conditions at terminal fatigue. This *Group* × *Color* interaction (*p* = 0.036) provides empirical evidence that the AR environment amplifies the physiological impact of the color–weight illusion. The shaded background in the APG panel denotes the mediated environment. *** denotes *p* < 0.001; ns, not significant.

Figure 7 presents the evolution of neuromuscular coordination strategies, quantified via the Co-Contraction Index (CCI), from baseline to exhaustion. The LMM analysis revealed a significant increase in CCI following the fatigue task (*F*_1,92_ = 329.7, *p* < 0.001), signaling a shift toward a protective joint-stiffening strategy as muscle capacity diminished.

Most importantly, a significant interaction between group and object color was confirmed (*F*_1,92_ = 4.5, *p* = 0.036), revealing a “reality gap” in physiological response. In the APG, the black stimulus induced a dramatically higher CCI at exhaustion (84.7 ± 25.4%) compared to the white stimulus (51.6 ± 13.0%). However, this discrepancy was markedly attenuated in the PRG (Table 1; BC: 76.2% vs. WC: 70.1%). Furthermore, a significant *Group* × *Time* interaction (*p* = 0.006) suggests that the AR environment significantly amplified the recruitment of antagonist muscles as physiological fatigue progressed, imposing a higher internal load on the musculoskeletal system.

## 4. Discussion

The findings of this study characterize the neuromuscular “reality gap” that emerges when the human motor system interacts with mediated visual cues in augmented reality (AR). Our primary discovery is that the AR environment significantly amplifies the physiological impact of the color–weight illusion (CWI), particularly under low-luminance (black) conditions. While the physical reality group (PRG) maintained relatively stable neuromuscular strategies regardless of object color, the augmented reality group (APG) exhibited a dramatic surge in the Co-Contraction Index (CCI) and an accelerated decline in median frequency (MDF). These results indicate that in the absence of high-fidelity haptic feedback, the central nervous system (CNS) overweights virtual visual stimuli, triggering an aggressive joint-stiffening strategy that increases the metabolic “bio-cost” of physically identical tasks [39,40].

The robust *Group* × *Color* interaction (*p* = 0.036) in the CCI provides empirical evidence for the “reality gap” in sensory integration. In the PRG, the impact of color was statistically negligible (*p* = 0.544, *BF*_10_ = 0.28), suggesting that in the physical world, the brain utilizes rich multi-sensory feedback to discount illusory visual cues. However, in the APG, the black stimulus induced a dramatically higher terminal CCI (84.7 ± 25.4%) compared to the white stimulus (51.6 ± 13.0%). This disparity aligns with recent research on mediated reality objects, which suggests that visual modifications to physical properties create persistent perceptual biases—such as the perceived weight of mediated sticks—that the CNS struggles to resolve when natural depth cues are limited by a head-mounted display [3,41]. As a result, the CNS treats the digital overlay as a high-reliability signal, transforming luminance into a physiological multiplier of motor-planning effort [42,43].

Furthermore, MDF values typically decrease as muscle fatigue increases [43,44]. Marico et al. [45] reviewed the use of surface EMG for muscle fatigue evaluation in biomechanics, noting an increase in signal amplitude and a shift towards a lower frequency in the signal power spectrum during the fatigue process. It is commonly observed that during prolonged or repetitive lifting, the EMG activity of the active muscle increases to compensate for the muscle’s reduced ability to generate force [46,47]. However, biceps and triceps muscles exhibit different behaviors under fatigue. The observed downward shifts in the MDF of the biceps brachii provide objective myoelectric evidence that visual illusions directly alter the manifestations of fatigue. The rapid decline in the APG-Black condition (a drop of ≈21.7 Hz) signifies accelerated spectral compression, which is highly consistent with the “augmented endurance” theory introduced by Ban et al. [2,10]. While foundational work primarily highlighted behavioral shifts in lifting repetitions, our spectral analysis identifies the physiological mechanism: the “heavier” black stimulus triggers a high-force motor program for a submaximal task. This leads to the premature recruitment and synchronization of high-threshold, fast-twitch motor units [44,48]. Recent studies further suggest that this disruption of the internal model of gravity in AR makes chromaticity a critical catalyst for physiological exhaustion [49].

A critical insight from this analysis is the physiological nexus between localized muscle fatigue (MDF) and joint-stiffening strategies (CCI). In the APG-Black condition, the rapid MDF decline in both the agonist (biceps) and antagonist (triceps) was closely coupled with the surge in co-activation. This relationship illustrates that high CCI is not merely a marker of stability but a driver of metabolic cost. According to optimal control theory, muscle co-contraction embodies uncertainty; when the brain perceives uncertainty in a virtual interface, it increases the mechanical impedance of the joint as a feedforward strategy for robust control [15,50]. This “impedance over-compensation” increases intramuscular pressure and reduces local tissue oxygenation, which directly drives the spectral shift in the MDF [45]. Consequently, the user’s arm is “fighting itself” to stabilize a perceived weight, resulting in a state where the metabolic “bio-cost” is no longer proportional to the physical torque required for the lift [51].

A vital observation for the HCI community is the decoupling of behavioral output and internal strain. Despite the extreme physiological cost observed in the APG-Black condition, participants maintained a relatively high repetition count (86.2 reps) to achieve “task parity” with the physical world. This suggests that evaluating AR systems solely through completion time or repetition counts can mask severe ergonomic risks, potentially leading to cumulative trauma disorders (CTDs) in long-term industrial settings [52]. These findings have significant implications for industrial assembly/disassembly (AD) and robotic-assisted rehabilitation [53,54]. In AD tasks, modulating virtual luminance can be used to manage worker fatigue and optimize joint stability. In clinical contexts, brightness-driven co-contraction offers a non-mechanical tool for strengthening coordination and joint stability training. Future AR systems integrating AI-driven digital twins could dynamically decode these physiological states (CCI/MDF) in real-time to personalize assistance and prevent injury before the onset of clinical fatigue.

### Limitations

However, this study has several limitations. First, the sample size was modest and unbalanced between groups. Although the linear mixed-effects model (LMM) framework helps address some constraints associated with unequal group sizes, the present design may still limit the precision and generalizability of the observed effects, particularly for interaction terms. In addition, because the cohort consisted primarily of young healthy adults, the findings should not be generalized to older populations without caution. A formal between-condition kinematic comparison was also not included; therefore, residual differences in movement execution across conditions cannot be completely excluded. Second, interactive methods in virtual reality do not always faithfully replicate real-world interactions, and the weight of virtual objects may not be conveyed accurately through visual cues alone [5,55]. Third, although the observed MDF and CCI changes are consistent with increased neuromuscular fatigue, the present design does not allow peripheral muscle fatigue to be distinguished from centrally mediated fatigue related to AR visual processing or visual strain. Moreover, no direct perceptual measures of the color–weight illusion were collected, such as subjective ratings of perceived heaviness, illusion strength, or visual fatigue. Accordingly, the present findings should be interpreted as physiological and behavioral correlates under chromatic manipulation rather than as a direct quantification of the perceived illusion itself. Future studies should recruit larger and more diverse samples and combine sEMG with perceptual and neurophysiological measures to distinguish peripheral and central contributions more directly.

## 5. Conclusions

This study investigated the neuromuscular “reality gap” induced by the color–weight illusion during manual lifting tasks in an augmented reality environment, specifically comparing the coordination strategies of the AR group (APG) and the physical reality group (PRG). Our experimental results indicate that low-luminance (black) virtual stimuli significantly amplify physiological strain in AR, triggering an aggressive joint-stiffening strategy in the APG characterized by a terminal Co-Contraction Index (CCI) and a significantly accelerated spectral decline in median frequency compared to the PRG. These results suggest that, when interacting with mediated reality objects, the central nervous system may overweight virtual visual cues relative to physical feedback, potentially increasing the physiological cost of the task under visuo-haptic uncertainty. The primary contribution of this work lies in identifying a critical “masking effect” where behavioral parties hide severe ergonomic risks, emphasizing the necessity for neuro-ergonomic assessment in AR design to prevent cumulative trauma in industrial settings. Furthermore, the findings offer a novel framework for future physiology-informed closed-loop systems, supported by AI-driven digital twins, to dynamically modulate virtual environments and optimize user safety and coordination training in real time.

## Figures and Tables

**Table 1:** Descriptive summary of physiological and behavioral performance.

Group	Condition	Repetitions	MDF_BB (Pre/Post) [Hz]	CCI (Pre/Post) [%]
ARG (*N* = 15)	BC	83.33 ± 25.84	90.1 ± 12.1/68.5 ± 8.8	14.0 ± 10.4/84.7 ± 25.4
	WC	103.20 ± 27.56	82.6 ± 10.6/71.4 ± 10.6	14.0 ± 7.8/51.6 ± 13.0
PRG (*N* = 10)	BC	98.33 ± 10.94	89.1 ± 10.0/66.7 ± 8.4	15.8 ± 16.4/76.2 ± 15.5
	WC	105.33 ± 13.12	91.4 ± 23.4/74.0 ± 13.1	10.6 ± 7.5/70.1 ± 5.1

Notes: Values are expressed as mean ± standard deviation (SD). MDF_BB: median frequency of the biceps brachii (primary agonist); CCI: Co-Contraction Index (biceps vs. triceps); Pre-FT: baseline measurements before the fatiguing task; Post-FT: terminal measurements at the point of exhaustion.

**Table 2:** Linear mixed-effects model results for key experimental indicators.

Metric	Metric	*df*	*F*	*p* Value	Partial *η*^2^
TotalReps	Group	1, 23.0	18.36	<0.001 ***	0.161
	Color	1, 96.0	1.76	0.188	0.018
MDF_BB	Color	1, 92.0	5.89	0.017 *	0.060
	Phase (Time)	1, 92.0	8.42	0.005 **	0.084
	Color × Time	1, 92.0	13.68	<0.001 *	**0.13** **0**
TotalReps	Group	1, 23.0	5.48	0.021 *	0.056
	Color	1, 92.0	18.26	<0.001 ***	0.165
	Phase (Time)	1, 92.0	329.67	<0.001 ***	0.782
	Group × Color	1, 92.0	4.50	0.036 *	**0.047**
	Group × Time	1, 92.0	7.87	0.006 **	0.079
	Color × Time	1, 92.0	18.88	<0.001 ***	0.170

Notes: * *p* < 0.05, ** *p* < 0.01, *** *p* < 0.001. Partial *η*^2^: Effect size, where 0.01, 0.06, and 0.14 represent small, medium, and large effects, respectively. Bold rows highlight the primary interaction effects supporting the study’s core hypotheses.

## Data Availability

The data that support the findings of this study are available from the corresponding authors upon reasonable request.

## Supplementary Materials

The following supporting information can be downloaded at: https://www.mdpi.com/article/10.3390/s26092575/s1, Figure S1: Representative band-pass-filtered sEMG traces (20–400 Hz) of the biceps brachii and triceps brachii under the black-color and white-color conditions, together with the corresponding RMS envelopes. This figure is provided to illustrate the preprocessing workflow and representative condition-related signal patterns.
