# Supplementary material for: Physiological Impact of Chromatic-Weight Illusions in Augmented Reality: A Comparative sEMG Analysis of Muscle Fatigue and Stability
Source: Sensors (Basel). 2026 Apr 22;26(9):2575. doi: 10.3390/s26092575 (PMC13165667; doi:10.3390/s26092575)
Supplement: Supplementary file 1 [file sensors-26-02575-s001.zip › sensors-4210054-supplementary.pdf]

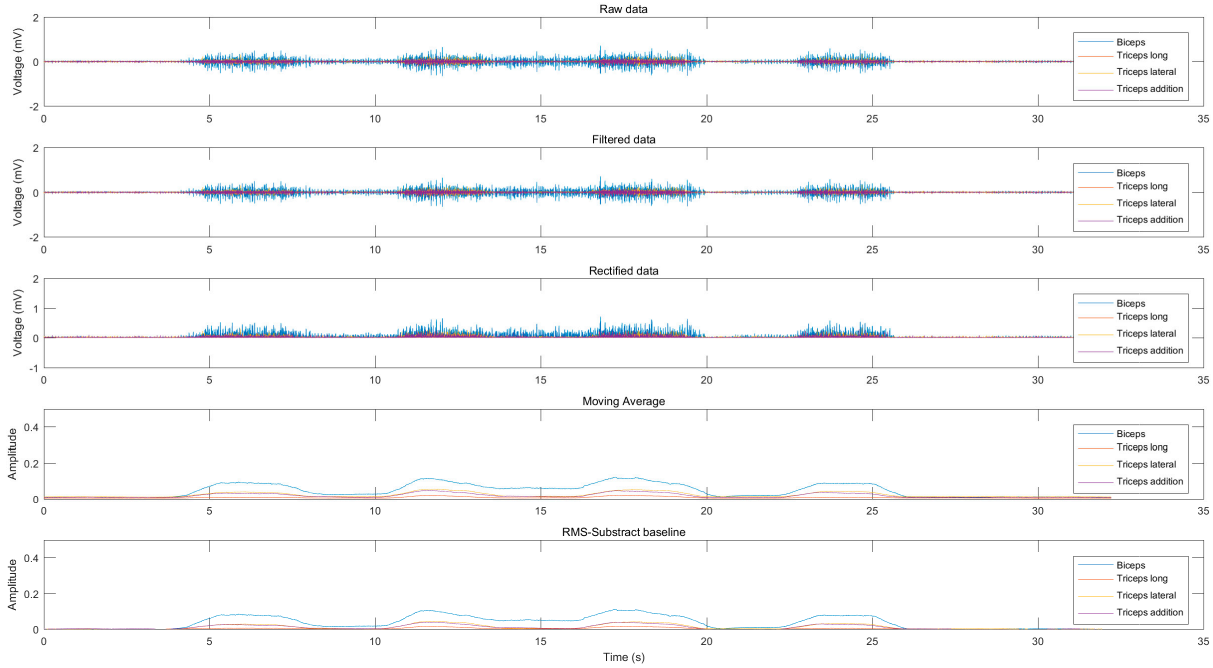

(a). Black condition

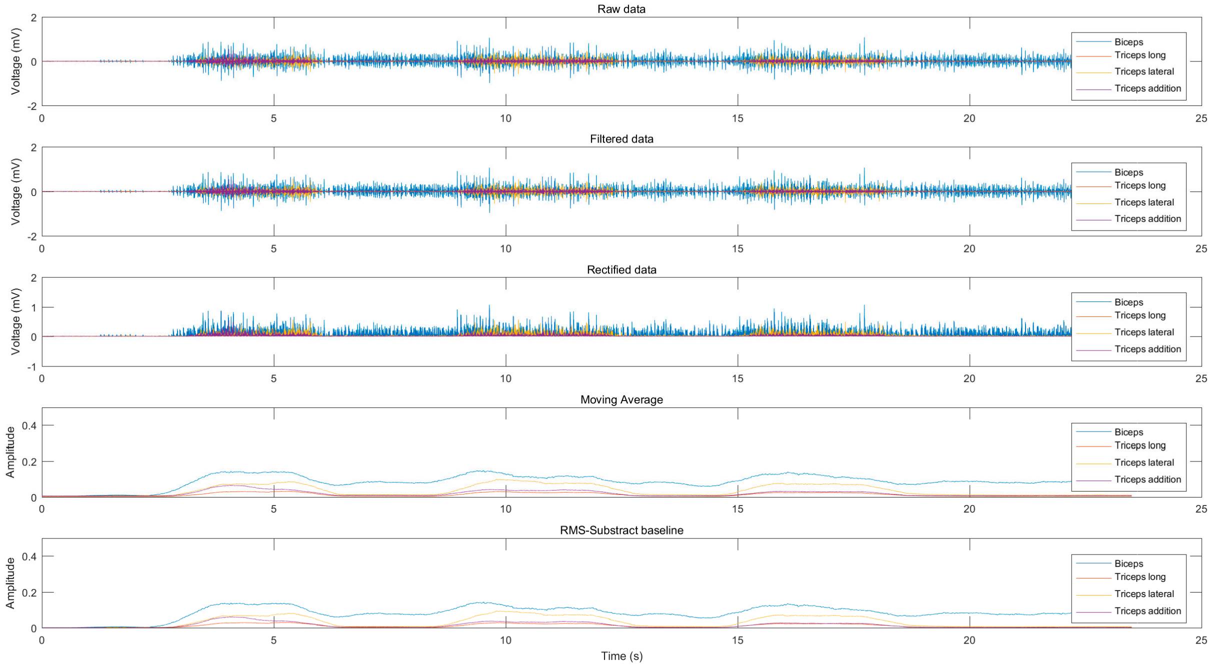

(b). White condition)

Figure S1. Representative band-pass-filtered sEMG traces (20–400 Hz) of the biceps brachii and triceps brachii under the black-color and white-color conditions, together with the corresponding RMS envelopes. This figure is provided to illustrate the preprocessing workflow and representative condition-related signal patterns.
